# Comparing Non-Invasive and Fluorescein Tear Break-Up Time in a Pre-Operative Refractive Surgery Population: Implications for Clinical Diagnosis

**DOI:** 10.3390/jcm14165794

**Published:** 2025-08-15

**Authors:** Rebecca Cairns, Richard N. McNeely, Mark C. M. Dunne, Raquel Gil-Cazorla, Shehzad A. Naroo, Jonathan E. Moore

**Affiliations:** 1 Cathedral Eye Clinic, Belfast BT1 2LS, UK; r.cairns@aston.ac.uk (R.C.);; 2 Optometry & Vision Science Research Group (OVSRG), College of Health and Life Sciences, Aston University, Birmingham B4 7ET, UK; m.c.m.dunne@aston.ac.uk (M.C.M.D.);; 3 Biomedical Sciences Research Institute, University of Ulster, Coleraine BT52 1SA, UK; 4 Clinical College of Ophthalmology, Tianjin Medical University, Tianjin 301700, China

**Keywords:** fluorescein break-up time (FBUT), non-invasive break-up time (NIBUT), dry eye disease (DED)

## Abstract

**Objectives:** Fluorescein break-up time (FBUT) is commonly used to assess tear film stability. However, the instillation of fluorescein destabilises the tear film, impacting validity and clinical applicability, while the subjective nature and variation in volume and concentration reduces repeatability. Non-invasive break-up time (NIBUT) offers an alternative method with less potential bias. Normal tear break-up time is conventionally accepted as 10 seconds (s); however, FBUT is expected to be lower than NIBUT. This study was designed to compare FBUT and NIBUT values in a pre-operative refractive surgery population, where diagnosis of dry eye disease may alter the risk–benefits ratio and contraindicate surgical procedure(s). Improved understanding of the relationship between these two methods will aid appropriate pre-operative patient counselling and consent. **Methods:** Data from consecutive participants presenting to a private ophthalmology clinic, for initial refractive surgery pre-operative assessment, were analysed. NIBUT and FBUT were performed. Paired and unpaired comparisons were made using the Wilcoxon signed-rank and Mann–Whitney U tests, respectively, and relationships with demographics were explored using Spearman’s rank correlation coefficient. **Results:** Median and interquartile range (IQR) for the first NIBUT was 12.5 s (7.0–18.0 s) and 14.2 s (9.4–18.0 s) for the right and left eyes, respectively. Median and IQR for the average NIBUT was 14.0 s (6.9–18.0 s) and 14.6 s (10.1–18.0 s) for the right and left eyes, respectively. Median and IQR for FBUT was 7 s (5–8 s) and 6 s (5–8 s) for the right and left eyes, respectively. There was a statistically significant difference between NIBUT and FBUT (*p* < 0.001). **Conclusions:** The findings suggest that the commonly used diagnostic threshold of 10 s cannot be uniformly applied to both FBUT and NIBUT, as FBUT systematically underestimates tear stability.

## 1. Introduction

Dry eye disease (DED) is a common condition that is experienced by approximately one in three adults [1]. Diagnosis is made when symptoms are accompanied by a loss of homeostasis of the tear film and/or ocular surface, which is evidenced by any clinical sign from tear film instability, tear hyperosmolarity, or the presence of ocular surface staining [2]. Its aetiology is multifactorial, including tear film instability, hyperosmolarity, ocular surface inflammation, ocular surface damage, or neurosensory abnormalities [3].

To assess tear film stability, fluorescein break-up time (FBUT) and non-invasive break-up time (NIBUT) are commonly used [4]. FBUT is typically performed at the slit lamp biomicroscope with broad beam cobalt and a Wratten filter applied, following instillation of sodium fluorescein from a moistened strip or minim to each eye, where the interval between the last blink and the appearance of the first visible area of break-up of the stained tear film is measured by the clinician [4,5,6]. NIBUT measures the time elapsed from the last blink to the first disruption of the specular reflection of either an illuminated grid or Placido rings from the tear film, and it is often automatically measured objectively from video recordings captured by videokeratoscopes with proprietary software [2]. FBUT, despite its widespread use, has notable limitations:

- Fluorescein instillation itself can destabilise the tear film, leading to artificially reduced break-up times [7,8,9,10,11,12].
- Variability in fluorescein volume and concentration affects test repeatability and inter-clinician reliability [2,7].
- FBUT measures the break-up of a dyed tear film, which may not accurately reflect natural tear film stability in non-stained conditions [2,7,9].

In contrast, NIBUT is a non-invasive alternative that eliminates the potential disruptive effects of fluorescein and may provide a more accurate representation of natural tear stability [6,13] despite being limited by the variability between devices [14]. However, there is no established consensus on whether FBUT and NIBUT can be used interchangeably, particularly in a pre-operative refractive surgery population. The 10 second (s) threshold for tear stability is commonly applied to both FBUT and NIBUT without differentiation [6,13], potentially leading to misclassification of patients into an abnormal tear film status with otherwise stable tear films. Given the critical role of tear film stability in refractive surgery outcomes, a more precise diagnostic standard is needed.

This study aims to compare FBUT and NIBUT in a pre-operative refractive surgery population, evaluating whether a single diagnostic threshold of 10 s is appropriate for both tests or whether both tests require different values. Understanding the relationship between two methods of measuring tear stability which currently have the same diagnostic cut-off value and where either method can be used in the diagnosis of DED [2] is critical to ensure consistent results from diagnostic criteria in clinical practice. This knowledge is important for all patients with respect to diagnosis and for developing a management plan and monitoring the condition and treatment efficacy.

It is especially important in the pre-operative assessment of patients presenting for refractive surgery, as a reduced TBUT, measured by either NIBUT or FBUT, may result in a diagnosis of pre-existing dry eye disease, thus altering the risk–benefit profile of the patient, suitability of specific surgical procedures, or indication of pre-operative management. Tear instability can also lead to irregular corneal topography and increased variability in keratometry readings [15,16], which in turn can compromise the accuracy of refractive surgery planning [16,17]. Understanding the relationship between these two methods will aid appropriate pre-operative patient counselling and consenting.

We hypothesise that FBUT systematically underestimates tear stability compared to NIBUT, which may necessitate test-specific diagnostic cut-off values for clinical decision-making.

## 2. Materials and Methods

Data from consecutive participants presenting to a private ophthalmology clinic for initial refractive surgery pre-operative assessment from 4 March 2024 to 21 June 2024 were analysed. All patients had refractive ametropia. Those with ocular comorbidities were not excluded, and these included amblyopia, corneal scar secondary to historic infection or foreign body, previous diagnosis of dry eye disease, epi-retinal membrane, early macular retinal pigment epithelium changes, and strabismus; prior ocular surgery included strabismus surgery, refractive laser surgery, and refractive lens exchange. All patients were asked to cease contact lens wear for at least two weeks prior to assessment and to refrain from instilling any topical medications for a minimum of two hours.

### 2.1. Patient Assessment

Comprehensive pre-operative assessment was performed, including, but not limited to, tear stability measures NIBUT and FBUT. NIBUT was the first clinical test performed, using the anterior segment optical coherence tomographer (AS-OCT) MS-39 (CSO, Firenze, Italy), with patients instructed to fixate on the target, blink twice, then stop. The proprietary software’s outputs the first NIBUT (time elapsed from preparatory blink to first blink) and average NIBUT (average time elapsed from previous blink to blink across video recording) were recorded. FBUT was performed following other clinical tests, where one drop of fluorescein sodium 1% minims was instilled into the lower fornix of each eye in random order and viewed through a slit lamp with broad beam cobalt and a Wratten filter, patients were instructed to blink three times then stop, and the time elapsed from the last blink until the first visible break was measured using a stopwatch. The average of 3 readings was recorded.

### 2.2. Statistical Analysis

Statistical analyses were performed using Microsoft Excel Version 16.99.2 (Microsoft, Redmond, WA, USA) and IBM SPSS Statistics Version 28 (IBM Corporation, 2019, IBM SPSS Statistics for Windows, Version 28.0.1.1, IBM Corp: Armonk, NY, USA).

A Kolmogorov–Smirnov test was conducted to test the normality of the data, and the data were deemed to be non-normally distributed (Kolmogorov–Smirnov test, all parameters *p* < 0.001).

Therefore, paired comparisons were made using the Wilcoxon signed-rank test, unpaired comparisons were made using the Mann–Whitney U test, and non-parametric relationships between variables were assessed using the two-tailed Spearman’s rank correlation coefficient.

To assess agreement between TBUT measurement techniques, Bland–Altman plots were constructed using the difference between paired measurements plotted against their median. The non-normal distribution of the data makes the median a more robust indicator of central tendency [18]. Non-parametric limits of agreement (LOA) were defined as the 2.5th and 97.5th percentiles of the differences, providing a distribution-agnostic estimate of agreement boundaries [19,20,21,22,23].

## 3. Results

Data was collected from 379 patients (52% male, mean age 48.1 (standard deviation (SD) = 13.6), and range 20–77 years) attending a private ophthalmology clinic in Belfast, Northern Ireland. Median and interquartile range (IQR) for the first NIBUT, average NIBUT, and FBUT for the right and left eyes are presented in Table 1. There was a statistically significant difference between the right FBUT and right first NIBUT (Z = −13.822, *p* < 0.001); right FBUT and right average NIBUT (Z = −15.887, *p* < 0.001); left FBUT and left first NIBUT (Z = −14.343, *p* < 0.001); and left FBUT and left average NIBUT (Z = −16.063, *p* < 0.001).

The difference between NIBUT and FBUT, the median of NIBUT and FBUT, and comparison of these values are displayed in Table 2.

**Figure 1:**
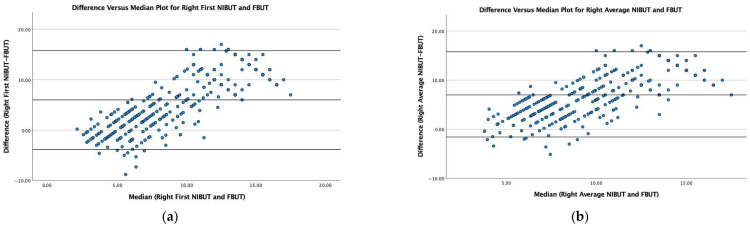

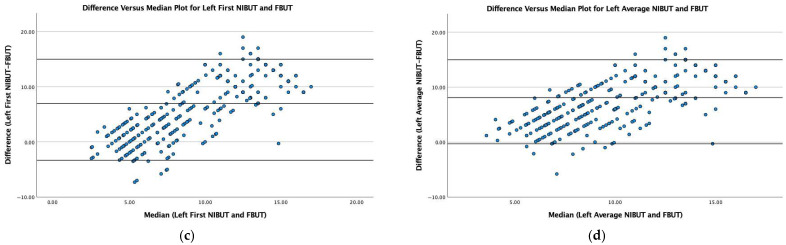
Difference (NIBUT-FBUT) versus median (NIBUT and FBUT) plot for FBUT and (**a**) right first NIBUT; (**b**) right average NIBUT; (**c**) left first NIBUT; (**d**) left average NIBUT. Each patient’s median (NIBUT and FBUT) value was plotted on the x-axis, and the difference between these values on the y-axis, enabling comparison of the two clinical tests, with evaluation of bias. Non-parametric lower and upper limits of agreement (LOA), the 2.5th and 97.5th percentiles, respectively, are shown for Difference (NIBUT-FBUT).

Figure 1 displays Bland–Altman plots for non-normally distributed data with Difference (NIBUT-FBUT) versus Median (NIBUT and FBUT) for both first and average NIBUT for the right and left eyes. There was a statistically significant correlation between NIBUT and FBUT for right (ρ = 0.822, *p* < 0.001) and left (ρ = 0.738, *p* < 0.001) eyes, and between NIBUT and FBUT for right (ρ = 0.799, *p* < 0.001) and left (ρ = 0.692, *p* < 0.001) eyes, with large effect sizes, where correlation coefficients of 0.1, 0.3, and 0.5 represent estimates of small, medium, and large size effects, respectively [24]. The Bland–Altman plots demonstrated a trend of increasing differences with higher median values, indicating proportional bias.

There was a statistically significant negative correlation between age and right first NIBUT, right FBUT, and left FBUT, as displayed in Table 3, with older participants exhibiting shorter tear break-up time (TBUT) with small to medium effect sizes. There was no statistically significant relationship between age and either right average NIBUT, left first NIBUT, or left average NIBUT.

There was a statistically significant relationship (*p* < 0.001) between sex and each measure of tear stability (right first NIBUT, right average NIBUT, left first NIBUT, left average NIBUT, right FBUT, and left FBUT) as presented in Table 3, with small to medium effect sizes, such that females have a shorter TBUT than males.

## 4. Discussion

The significant discrepancy found between FBUT and NIBUT values suggests that a single diagnostic cut-off value, such as the commonly used 10 s [6,13], cannot be applied to both tests. This is in line with other studies, which found mean values of 7.6 ± 10.4 s for FBUT and mean differences of 3.7 ± 12 s between NIBUT and FBUT [25] and 8.6 s for FBUT and 10.9 s for NIBUT [7].

The relationship between NIBUT and FBUT is contentiously reported in the literature, with several reporting that they are equivalent values [26,27,28] and others reporting a significant difference inverse to this study with FBUT longer than NIBUT [27,29,30,31]. Other publications report, as confirmed by this study, that the values obtained by both tests are significantly different, with FBUT shorter than NIBUT [7,9,10,11,14,25,32,33,34,35,36]. These conflicting reports, where FBUT is longer than NIBUT, may be due to a difference in methodology. For example, some studies measure tear break-up time in real time using a stopwatch, as in the current study, while other use video recordings. Additionally, the volume of fluorescein instilled can vary significantly. Some studies use a controlled volume (e.g., 2 µL) of fluorescein instilled [31], whereas this study used a standard minim drop, (25–75 µL [37]), reflecting a real-world clinical environment where measuring a more restricted volume is not practical. However, other studies have found no difference in FBUT values for normal individuals (or those with aqueous deficient DED) with different instillation volumes of fluorescein [4]. Alternative reasons for this disparity may be due to the subjective nature of FBUT or the different instruments used to measure NIBUT that have been shown not to be equivalent [14,29,33,38,39,40]. Some have found that NIBUT is longer than FBUT independent of the instrument used [33].

The non-parametric Bland–Altman difference-versus-median plots (Figure 1) have each patient’s median (NIBUT and FBUT) value plotted on the x-axis, and the difference between these two values plotted on the y-axis, with non-parametric lower and upper limits of agreement (LOA), the 2.5th and 97.5th percentiles, respectively, for Difference (NIBUT-FBUT) on the y-axis. This enables comparison of the two clinical tests, with evaluation of bias. Figure 1 illustrates less variability between NIBUT and FBUT with shorter tear break-up time (TBUT) and increasing difference between NIBUT and FBUT measurements as the median increases. This demonstrates a systemic divergence between methods at higher measurement values, where agreement between FBUT and NIBUT techniques diminishes as the magnitude of measurement of tear stability increases. This heteroscedasticity (i.e., non-uniform scatter of data around the regression line) confirms that FBUT and NIBUT measures of tear stability are not directly clinically comparable.

These results reinforce the appropriateness of median-based analysis and non-parametric limits of agreement (LOA) for non-normally distributed data [19,20,21,22,23]. Correlation analysis alone may conceal such bias, underscoring the importance of visual and distribution-aware methods.

This confirms previous reports of better agreement between NIBUT and FBUT for shorter TBUT [10,14]. It has also been reported there is less heteroscedasticity for both NIBUT and FBUT [14,36,41] for shorter TBUT.

It has been reported that instillation of fluorescein destabilises the tear film, thereby reducing TBUT [7,9,10,11,14,38,42]. However, this is contested with some authors reporting no change in TBUT with fluorescein instillation [28,43] and others reporting an increase in TBUT with instillation [7,8,12,14,29], some of whom find this increase in TBUT with fluorescein plateaus at volumes of 2.7 µL [12] or 7 µL [10]. The use of minims does control the concentration, 1% in this study, and eliminates the effects of any excipients in the saline solution used to moisten a fluorescein strip. Other groups using the minimum amount of fluorescein by shaking a wetted fluorescein strip to remove excess before instillation had similar results to this study.

Notably, in the normal pre-operative cohort, the median value was 12.5–14.6 s for NIBUT and 6–7 s for FBUT, confirming the hypothesis of this study that these two values are not equitable, a value of >10 s cannot be considered normal and <10 s used as a diagnostic criterion for DED for both methodologies. A diagnostic criterion of 5.3 to 6.0 s has been suggested for FBUT [4].

The study cohort included patients with refractive ametropia seeking refractive surgery, and it is therefore not representative of the general population. Participants with ocular comorbidities were not excluded from analysis, as the aim of this study was to examine the relationship between NIBUT and FBUT in patients presenting to a private ophthalmology clinic for initial refractive surgery pre-operative assessment. Real-world clinical data, while limited in its generalisability by its geographic location and associated demographics, is valuable as it presents insight into typical clinical practice, which can be applied to those with similar patient cohorts and help identify current knowledge gaps that directly impact patient care for future research.

This study investigated the impact of age on TBUT and found a significant negative correlation with age and right first NIBUT (*p* = 0.046), right FBUT (*p* < 0.001), and left FBUT (*p* < 0.001), confirming previous studies [44,45] while also being in line with the known increased prevalence of DED with age [46]. However, this relationship was not observed for the right average NIBUT, left first NIBUT, or left average NIBUT, similar to other studies [13,47]. A larger sample size would be required to reach a high enough statistical power given the small effect size.

The impact of sex on TBUT was also examined, and a statistically significant relationship was found for all measures of TBUT (*p* < 0.001), with females exhibiting a shorter TBUT than males. This confirms previous studies [44,45,48], which similarly reported a greater incidence of DED in females than males [46,49], but contrasts others which found no relationship [13,47].

The statistically significant relationship between, and positive correlation of, difference-versus-median plots shows that FBUT underestimation increases with TBUT, such that the longer the TBUT, the greater the discrepancy between NIBUT and FBUT. FBUT has previously been observed as being statistically significantly shorter than NIBUT [10,14,27,29,33,38]. However, the values from one NIBUT device are not directly interchangeable with those from another [14], and this is the first study using the MS-39 with a large pre-operative cohort [26], confirming the dependency [10,14,32].

The authors hypothesise that this dependency is due to the instilled irritant fluorescein sodium causing reflex tearing, diluting the lipid layer and therefore reducing tear stability [32,33], with individuals who have a thicker lipid layer and more stable tear film initially, as measured by NIBUT being more affected. Similarly, FBUT may also be affected by glare-induced lacrimation due to infrared exposure [10]. Another posited reason for this discrepancy is due to the differences in test design, with FBUT measuring the initial disintegration of the fluorescein-stained aqueous layer while NIBUT measures the following break-up of the lipid layer by monitoring disturbance of reflected Placido disk images from the anterior ocular surface [32,33,35]. Another theory is that FBUT, as a measure of the whole tear film, is influenced by volume changes caused by its own instillation whereas NIBUT, as a measure of surface stability, is not [14].

The NIBUT diagnostic threshold for DED may be ≤10 s [9] while <5 s [50] and 5.3 to 6 s [4] has been suggested for FBUT. While defining diagnostic thresholds was beyond the scope of this study, the median values obtained for NIBUT and FBUT from this pre-operative population corroborate these. Further research is required to determine the mechanism by which fluorescein impacts TBUT and to determine normative values and diagnostic cut-off values for DED for FBUT and NIBUT that are specific to each methodology.

## 5. Conclusions

This study demonstrates that FBUT consistently underestimates tear stability compared to NIBUT, emphasising the need for test-specific diagnostic thresholds. The widely accepted 10 s cut-off is inappropriate for FBUT and should be revised downward to avoid over-diagnosis of tear instability. Given its stability and non-invasive nature, NIBUT may be the preferred method for pre-operative refractive surgery evaluations.

Future research should focus on establishing standardised diagnostic thresholds for both FBUT and NIBUT, incorporating a broader patient population and comparing different measurement techniques to optimise clinical decision-making.

## Figures and Tables

**Table 1:** Median and interquartile range for the first NIBUT, average NIBUT, and FBUT.

	First NIBUT	FBUT	Average NIBUT
Right	12.5 s (7.0–18.0)	7 s (5–8)	14.0 s (6.9–18.0)
Left	14.2 s (9.4–18.0)	6 s (5–8)	14.6 s (10.1–18.0)

**Table 2:** Median and interquartile range for the difference between, and the median of, NIBUT and FBUT. Wilcoxon signed-rank paired comparisons (Z) and two-tailed Spearman’s correlation coefficient (ρ) and respective *p* values are shown for the difference (NIBUT-FBUT) compared to the median (NIBUT and FBUT).

	Median Difference (NIBUT-FBUT)	Wilcoxon Signed-Rank (Z) Difference Versus Median	Spearman’s Correlation Coefficient (ρ) Difference Versus Median	Median (NIBUT and FBUT)
Right First NIBUT	6 s (1.5–11.0)	Z = −14.099 *p* < 0.001	ρ = 0.821	9.4 s (6.1–13.5)
			*p* < 0.001	
Right Average NIBUT	7 s (3.9–11.0)	Z = −13.235 *p* < 0.001	ρ = 0.761	10.3 s (7.4–13.5)
			*p* < 0.001	
Left First NIBUT	7 s (1.7–11.0)	Z = −13.678 *p* < 0.001	ρ = 0.789	10 s (6.25–13.1)
			*p* < 0.001	
Left Average NIBUT	8.1 s (4.4–11.0)	Z = −12.692 *p* < 0.001	ρ = 0.713	10.5 s (7.8–13.4)
			*p* < 0.001	

**Table 3:** Correlations with first NIBUT, average NIBUT, and FBUT for both right and left eyes; using the Spearman’s rank correlation coefficient (ρ) for investigating the correlation with age; and the Mann–Whitney U test (Z) for investigating the correlation with sex.

	Age	Sex
Right First NIBUT	ρ = −0.104 * N = 366 *p* = 0.046	Z = −5.585 *
		N = 366
		*p* < 0.001
		r = −0.292
Right Average NIBUT	ρ = −0.068 N = 366 *p* = 0.197	Z = −4.924 *
		N = 366
		*p* < 0.001
		r = −0.257
Left First NIBUT	ρ = −0.02 N = 367 *p* = 0.971	Z = −5.759 *
		N = 363
		*p* < 0.001
		r = −0.302
Left Average NIBUT	ρ = 0.04 N = 367 *p* = 0.935	Z = −4.750 *
		N = 363
		*p* < 0.001
		r = −0.249
Right FBUT	ρ = −0.219 * N = 356 *p* < 0.001	Z = −6.633 *
		N = 349
		*p* < 0.001
		r = −0.355
Left FBUT	ρ = −0.257 * N = 358 *p* < 0.001	Z = −6.968 *
		N = 351
		*p* < 0.001
		r = −0.372

* Indicates statistically significant correlations.

## Data Availability

The raw data supporting the conclusions of this article will be made available by the authors on request.

## Abbreviations

The following abbreviations are used in this manuscript:

FBUT	Fluorescein break-up time
NIBUT	Non-invasive break-up time
TBUT	Tear break-up time
s	Seconds
SD	Standard deviation
IQR	Inter-quartile range
DED	Dry eye disease
AS-OCT	Anterior segment optical coherence tomographer
Z	Wilcoxon signed-rank
Z	Mann–Whitney U
ρ	Spearman’s correlation coefficient
